# Fisetin—In Search of Better Bioavailability—From Macro to Nano Modifications: A Review

**DOI:** 10.3390/ijms241814158

**Published:** 2023-09-15

**Authors:** Joanna Szymczak, Judyta Cielecka-Piontek

**Affiliations:** Department of Pharmacognosy and Biomaterials, Poznan University of Medical Sciences, Rokietnicka 3, 60-806 Poznan, Poland; jszymczak@ump.edu.pl

**Keywords:** fisetin, drug delivery, phytoconstituents, bioavailability

## Abstract

As secondary plant metabolites, polyphenols are abundant in fruits and vegetables. They are in high demand because of their many health benefits. However, their low bioavailability makes them complex compounds to use for therapeutic purposes. Due to the limited solubility of phytocompounds, dietary supplements made from them may only be partially effective. Such molecules include fisetin, found in strawberries, and have shown great promise in treating Alzheimer’s disease and cancer. Unfortunately, because of their limited water solubility, low absorption, and poor bioavailability, the assistance of nanotechnology is required to allow them to fulfil their potential fully. Here, we provide evidence that nanodelivery methods and structure modifications can improve fisetin bioavailability, which is linked to improvements in therapeutic efficacy. An open question remains as to which nanocarrier should be chosen to meet the abovementioned requirements and be able to enhance fisetin’s therapeutic potential to treat a particular disease.

## 1. Introduction

Plants have always played significant roles in human life due to their content of compounds that are beneficial to health. Phytocompounds still hold great interest, as evidenced by numerous studies on their properties, and the availability of dietary supplements—especially polyphenols—as specialized secondary plant metabolites attracts attention owing to their health-promoting potential. The beneficial effects of these compounds are most often associated with the modulation of multiple molecular mechanisms. Molecular structure, particularly the presence of hydroxyl groups, determines antioxidant properties. Thus, polyphenols can be used as weapons to fight against diseases caused by oxidative stress, e.g., cardiovascular disorders or cancers [1,2]. An interesting molecule belonging to the class of polyphenols is fisetin, for which many activities have been proven, including antioxidant activity and neuroprotective, anti-inflammatory, and senolytic effects [3,4,5]. The factors limiting its biological impact on organs and membranes in the human body are its low solubility in water and body fluids and its low absorption in the gastrointestinal environment [6]. Therefore, novel delivery systems and structure modifications are introduced to overcome its poor bioavailability. Thus, this review aims to analyze studies reporting on improving fisetin’s bioavailability and enhancing its therapeutic potential. In particular, this review focuses on using new nanodelivery systems such as polymer nanoparticles, various lipid vesicles, complexes with cyclodextrins, systems based on pre/probiotics, nanocrystals, and semi-solid systems.

## 2. Fisetin

**Figure 1:**
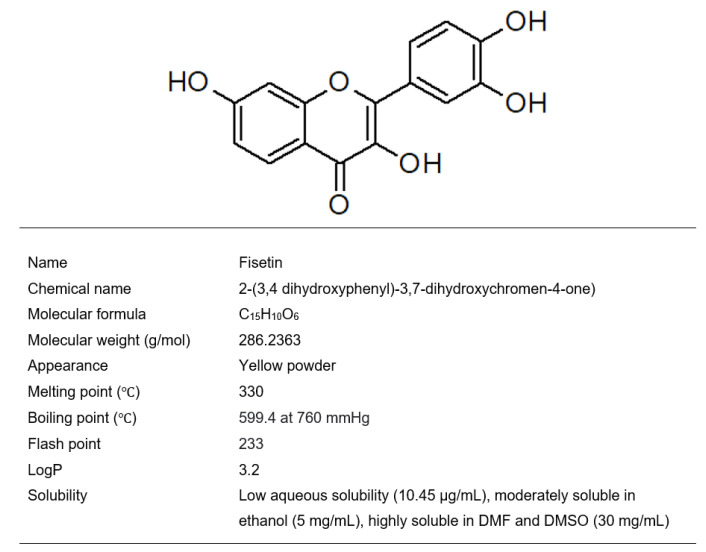
Molecular structure and physicochemical properties of fisetin.

Fisetin (3,3′,4′,7-tetrahydroxyflavone) (Figure 1) is a yellow-colored compound belonging to the polyphenols in a subgroup of the flavonoid class. This bioactive flavonol is present at a concentration in the range of 0.1–160 µg/g in various fruits and vegetables such as strawberries, apples, persimmons, lotus roots, onions, grapes, kiwis, peaches, tomatoes, and cucumbers. What is worth noting is that strawberries are the best dietary source of fisetin (160 µg/g) [7].

Fisetin has attracted attention due to its interesting biological activity, including anti-tumor and anti-inflammatory activity and other pharmacological effects. The possible mechanisms of action in various diseases are summarized in Table 1. Most research focuses on the potential use of fisetin as an anticancer drug. The anti-tumor effect of fisetin is associated with the regulation of phosphoinositide-3-kinase (PI3K), protein kinase B (AKT), serine-threonine protein kinase (mTOR), and Wnt/β-catenin-driven pathways, as well as the activation of the tumor-necrosis-factor-related apoptosis-inducing ligand (TRAIL), leading to apoptosis and the inhibition of angiogenesis (downregulation of vascular endothelial growth factor (VEGF) expression). This results in autophagic and apoptotic cell death and consequently leads to the inhibition of tumor growth [8]. Fisetin also demonstrates anti-inflammatory effects through the control of the signaling pathway of the nuclear factor kappa-light-chain-enhancer of activated B cells (NF-κB), the inhibition of nitric oxide, and the production of proinflammatory cytokines, e.g., tumor necrosis factor-alpha (TNF-α), interleukin -1β (IL-1β), IL-6, and IL-8 [9]. It offers promising prospects in terms of treating inflammatory diseases, e.g., hepatic ischemia/reperfusion injury [10]. In addition, fisetin exerts antioxidant properties via interactions with redox-related signaling pathways (NF-κB, mitogen-activated protein kinases (MAPK), nuclear factor erythroid 2-related factor 2 (Nrf2), and PI3K/AKT). It has the ability to scavenge free radicals. Through the expression of antioxidant enzymes, it can protect cell components from oxidative damage [3]. Other studies showed that fisetin could be a promising drug for treating neurological disorders such as Alzheimer’s disease, Parkinson’s disease, and traumatic brain injury [11,12,13]. The pharmacological effect of fisetin on osteoporosis was also demonstrated in many in vitro and in vivo studies [14]. The beneficial effect was obtained through osteoblast activation and the inhibition of bone loss. Another unique property of fisetin is its senotherapeutic activity. The reduced expression of senescence markers in different organs is attributed to the anti-aging activity of fisetin [5]. Studies have shown that fisetin can inhibit vascular smooth muscle cell senescence by increasing the phosphatase and tensin homolog deleted on chromosome 10 (PTEN) and decreasing the levels of the mammalian target of rapamycin complex 2 (mTORC_2_) protein [15]. Therefore, fisetin has the potential to be used to treat age-related pathologies in elderly patients.

## 3. Bioavailability and Bioefficacy

Although fisetin has a wide range of pharmacological potential, it has limitations such as low bioavailability (44.1%), poor water solubility (10.45 g/mL), and high lipophilicity (logP 3.2) [27]. According to a pharmacokinetic study, fisetin is absorbed quickly and undergoes phase II biotransformation into sulfates and glucuronides. The maximal concentration of fisetin (2.53 g/mL) was attained after 15 min of i.p. treatment (223 mg/kg) [28]. Because of this, frequent dose administration is required to ensure therapeutic efficacy. The bioavailability enhancement will enable dosage reduction and prevent the onset of unwanted side-effects. However, it is important to note that, in a mouse model, fisetin was found to be rapidly distributed in the brain vesicles and parenchyma following oral and intraperitoneal injection [29]. Considering its potential for use in neuroprotection, penetration of the brain barrier is essential for reaching the site of action. Considering the abovementioned limitations, there is an urgent need to improve its low bioavailability. As a factor that affects bioefficacy, bioavailability is necessary to produce an intended biological response. The biological effect cannot be noticed when it is at a low level. Thus, introducing structure modifications and using nanocarriers can raise the possibility of using fisetin’s pharmacological potential to treat various diseases.

## 4. Nanoparticle-Based Delivery Systems

Nanoparticles (NPs) have sizes ranging from 10 to 1000 nm. They are made of various substances, such as polymers, lipids, and dendrimers. Their properties and potential applications are determined by their physicochemical characteristics [30]. Nanoparticles have large surface areas that interact with solvents, thus increasing the solubility of bioactive compounds. This has been shown using the Noyes–Whitney equation [31]. This makes them suitable nanocarriers for use in delivery systems of poorly soluble drugs to enhance drug solubility, bioavailability, and bioefficacy.

### 4.1. Polymeric Nanoparticles

Fisetin has anti-tumor activity when added to polymeric nanoparticles. In the study by Feng et al., fisetin was encapsulated in poly(lactic acid) (PLA) using the spontaneous emulsification solvent diffusion method [32]. The researchers chose the optimal preparation parameters of a fisetin/PLA ratio of 1:10, an oil-to-water ratio of 1:7, an acetone/ethanol ratio of 3:3, and 0.5% Poloxamer 188. The developed formulation had an encapsulation effectiveness of 90.35 ± 2.34% and a particle size of 226.85 ± 4.78 nm. According to in vivo anticancer experiments performed on a 4T1 breast cancer model, fisetin nanoparticles had greater anti-tumor activity to free fisetin. The fisetin-loaded PLA also reduced tumor volume to 64.7%.

The addition of monomethyl poly(ethylene glycol)-poly(ε-caprolactone) (MPEG-PCL) to fisetin is another method that illustrates a means of boosting bioefficiency [33]. After the i.v. administration of fisetin-loaded copolymer treatment, the volume and weight of the tumor were decreased in a mouse LL/2 tumor model. The nanoparticles showed no toxicity to other mice organs. In vivo experimentation confirmed the safety of MPEG-PCL polymer application, with a cell survival at a level higher than 76%. 

When encapsulated in poly(vinylpyrrolidone) (PVP) nanoparticles, fisetin demonstrated superior antiproliferative action than free fisetin, and the growth inhibition of MDA-MB-231 breast cancer cells increased with a change in cell survival rate from 35 and 62%, respectively. The release of fisetin from the polymeric carrier was 2 times higher than that of free fisetin after 24 h. The improved dissolution rate and solubility could result from the decreased particle size and increased surface area of formulated nanoparticles [34].

Other researchers investigated how the gastrointestinal environment affected the release of fisetin from hydrophobic and more hydrophilic polymeric nanocarriers. To accomplish this, Sechi et al. created fisetin-loaded nanoparticles containing poly(D, L-lactic-co-glycolic acid)-block poly(ethylene glycol) carboxylic acid (PLGA-PEG-COOH) and poly(-caprolactone). The particle size ranged from 140 to 200 nm with an encapsulation efficacy of 70 to 82%. In simulated gastrointestinal conditions, fisetin formulations demonstrated regulated release. Fisetin was protected by a polymeric carrier from acidic stomach juice, resulting in an initial burst and reduced release at an acidic pH (<15% in all cases). In basic pH conditions, fisetin release began to increase gradually. The polymeric formulation’s composition significantly affected the release and could thus influence bioavailability. The majority of hydrophilic nanoparticles containing PLGA-PEG-COOH showed a 70% release of fisetin after 7 h, while particles containing hydrophobic PCL achieved the same result only after 24 h [35].

Another team of researchers examined the permeability of fisetin-loaded poly(lactic-co-glycolic acid) (PLGA) and polyvinyl alcohol (PVA) nanoparticles across various gut regions to determine their bioavailability using an everted gut sac permeability test. An increase in the permeation was observed, especially in the duodenum region, where it was 4.9 times greater than suspension. In addition to having improved intestinal permeability, the fisetin nanoparticles remained stable for 60 days, with no signs of aggregation or changes in particle size. The release experiments revealed that the release of fisetin from nanoparticles was higher than the level of free fisetin in all pH ranges (pH 1.2, 6.8, and 7.4) throughout the 24-hour period [36].

When discussing bioavailability, it is also important to note the pharmacokinetic studies’ results on fisetin encapsulated in poly(lactic acid) (PLA). After the administration of i.v. treatment to rats, fisetin nanoparticles showed increased half-life (3.42 h) and AUC (19.28 μg/mL) compared with free fisetin (t_1/2_ = 1.84 h, 8.31 μg/mL). The results may be explained by fisetin’s prolonged release from polymer nanoparticles (over 80% of the drug is released after four days) and by the bypassing of the phagocyte system due to the negatively charged surface [32].

### 4.2. Polymeric Micelles

**Figure 2:**
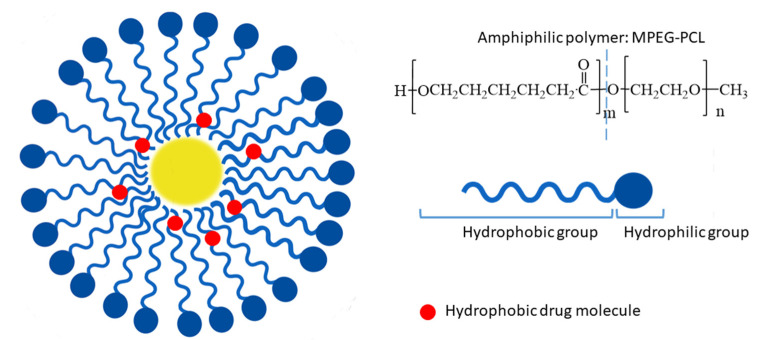
Schematic representation of polymeric micelle prepared of amphiphilic polymer MPEG-PCL; methoxy poly(ethylene glycol)-poly(ε-caprolactone) (MPEG-PCL).

To improve the therapeutic effect on colon cancer, fisetin was loaded into MPEG-PCL polymeric micelles via self-assembly (Figure 2). The particle size of the prepared micelles was 22 ± 3 nm with a polydispersity index of 0.163 ± 0.032 and an encapsulation efficiency of 98.53 ± 0.02%. The results obtained from the MTT assay demonstrated the enhanced cytotoxicity of fisetin micelles against CT26 cells compared to free fisetin. Furthermore, as the flow cytometric histograms exhibited, the cellular accumulation of fisetin polymeric micelles was higher after 4 h of incubation than that of free fisetin. Additionally, the suppression of tumor growth and prolonged survival were observed after the treatment of mice bearing tumors with fisetin micelles. The mice’s life was extended by 17 days in the case of using fisetin micelles compared to treatment with fisetin alone [37].

Other researchers prepared fisetin-loaded MPEG-PCL polymeric micelles with similar parameters as seen in the abovementioned example. The antitumor efficacy of fisetin formulation was determined using a mouse model with SKOV3 ovarian cancer cells. Prepared micelles exhibited great anticancer activity and apoptosis induction with 1.3-times-stronger tumor growth inhibition than free fisetin [38].

To improve the anticancer efficacy of fisetin against breast cancer cells, Wang et al. designed fisetin-loaded polymeric micelles using poly(lactic acid) conjugated with D-α-tocopheryl polyethylene glycol 1000 via a solvent evaporation method. In vitro cytotoxicity assay results showed that fisetin polymeric micelles were more toxic than others to MCF-7 breast cancer cells. The IC_50_ value for encapsulated fisetin was 2.93 times lower than the value calculated for free fisetin. Furthermore, the prepared formulation exhibited greater induction of apoptosis after 24 and 48 h (ca. 20% and 42% respectively) and significantly reduced tumor growth in the mice [39].

Polymeric micelle formulation for the treatment of breast cancer cells was also prepared. Pawar et al. loaded fisetin in pluronic F127 copolymers conjugated, with folic acid acting as a targeting ligand, using a thin-film hydration method. The prepared micelles displayed a spherical shape with a diameter size of 103.2 ± 6.1 nm, encapsulation efficiency of 82.50 ± 1.78%, and negative zeta potential (−26.7 ± 0.44 mV). During in vitro anticancer activity studies, fisetin micelles induced pronounced effects on human breast cancer cells (MCF-7), with the GI_50_ (growth inhibition of 50%) value decreasing by 65.737% compared to free fisetin. In vitro studies showed sustained release of fisetin from micelles, which increased steadily up to 120 h. In a slightly acidic medium (pH 5.3) mimicking the cancer cells’ environment, the release of fisetin was enhanced after 5 days (80%) compared to the release observed at pH 7.4 (50%). Moreover, encapsulation in micelles improved fisetin’s bioavailability, resulting in 1.8-, 2-, and 6.3-fold increases in C_max_, t_1/2,_ and AUC. The prepared fisetin formulation showed enhanced anticancer activity, reduced systemic toxicity, and better bioavailability than free fisetin [40].

### 4.3. Human Serum Albumin Nanoparticles

Human serum albumin nanoparticles (HSA-NPs) are among several polymeric formulations developed for drug delivery. They have gained considerable attention due to advantages such as biocompatibility, biodegradability, and good toleration after application [41]. Fisetin-loaded HSA nanoparticles were prepared using a desolvation method. The developed spherical particles had an average size of 220 ± 8 nm and a good encapsulation efficiency of 84%. The in vitro release study showed a biphasic release pattern, with the release rate increasing along with the ionic strength of the solution. Similar to formulations developed earlier, a quick initial release was seen, followed by a slowdown. After 72 h, the total release value reached ca. 75%. Encapsulation in HSA NPs resulted in decreasing activity of the scavenging DPPH radical, with an inhibition rate of ~51% compared to free fisetin (~85%). Moreover, as demonstrated in vivo studies on MCF-7 cells, fisetin nanoparticles showed stronger cytotoxic activity against breast cancer cells than fisetin alone [42].

### 4.4. Nanoemulsions

**Figure 3:**
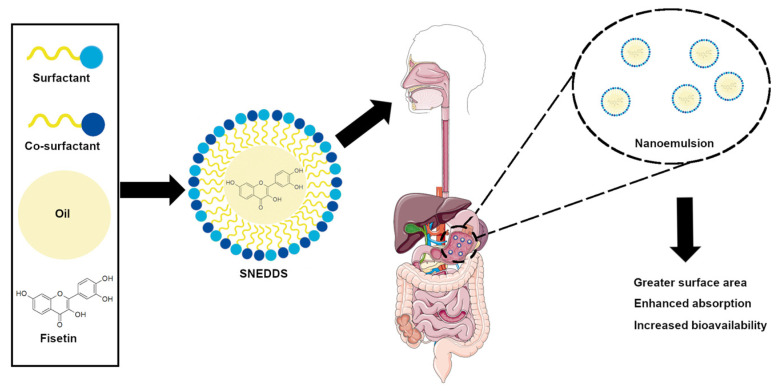
Schematic diagram of SNEDDS preparation with fisetin for oral administration; SNEDDS: self-nonoemulsifying drug delivery system.

Nanoemulsions are classified as colloidal dispersions, which consist of two immiscible liquids. The dispersed phase and the dispersing medium are mixed to form a single phase with the addition of an emulsifying agent, i.e., surfactant or co-surfactant. As a result, a transparent dispersion of oil in water (o/w) or water in oil (w/o) is obtained, with a particle size in the range of 20–200 nm [43]. More recently, self-nonoemulsifying drug delivery systems (SNEDDS) have become a new kind of nanoemulsions that can be used as nanocarriers. These self-emulsifying formulations are an anhydrous nanoemulsions consisting of oil, a surfactant, a co-surfactant, and a drug molecule. An oil-in-water (o/w) nanoemulsion is formed upon dilution in the aqueous phase. This process occurs when SNEDSS is administered orally, causing nanoemulsion in the stomach. The smaller particle size in the nano range provides a greater surface area for absorption and thus increases drug bioavailability (Figure 3) [44,45,46]. Considering these advantages, researchers prepared a self-nanoemulsyfying drug delivery system, using fisetin to enhance its solubility, permeability, and bioefficacy.

Kumar et al. prepared SNEDSS with fisetin using castor oil, Lauroglycol FCC, Tween 80, and Transcutol P. The developed formulation had a droplet size of 154 nm. The fisetin-SNEDSS showed increased dissolution rates compared to free fisetin in different media. It was 11.03-, 3.55-, and 7.9-fold higher in water, a pH 1.2 HCl buffer, and a pH 6.8 phosphate buffer, respectively. Moreover, there was no significant change in physicochemical characteristics, e.g., droplet size, when diluted in media of different pH. This is significant when the drug carrier is prepared for oral administration and must travel through changing pH levels in the gastrointestinal tract after swallowing.

To evaluate cell cytotoxicity, the MTT assay was used. The results revealed that fisetin in SNEDDS is less toxic (89.05% viable cells) due to decreased interaction with the cells than free fisetin (10.08% viable cells). During in vitro cell line permeability studies, fisetin-loaded SNEDDS exhibited 3.79-fold higher permeation and 0.79-fold lower drug excretion than free the fisetin molecule after 5 h. The preparation of SNEDDS with fisetin allowed the researchers to obtain a stable formulation with enhanced permeability in cell lines [47].

The same research group tested the pharmacokinetic parameters of the fisetin-loaded SNEDDS with a droplet size of 154 ± 8.5 nm and a drug loading of 100 ± 0.92%. The pharmacokinetic study of the formulation was conducted after oral administration of 20 mg/kg to rats. The use of fisetin in SNEDDS exhibited a 3.7-fold increase in C_max_ and higher T_max_ (30 min) compared to the use of fisetin alone (T_max_ = 15 min). The improved oral bioavailability can be attributed to enhanced system absorption of fisetin in nanoemulsion. The developed formulation was also subjected to activity studies against rotenone-induced Parkinson’s disease in rats. After administering two doses orally, i.e., 10 mg/kg and 20 mg/kg, behavioral studies were conducted weekly during the 35-day test. The fisetin–SNEDDS combination reduced the negative effect of rotenone-induced behavioral alternations in the rats, such as locomotor or muscle coordination activity and catalepsy. Biochemical parameters also improved. The fisetin formulation showed antioxidant activity, reducing the level of TNF-α and IL-6 and improving the concentration of antioxidant enzymes, i.e., it reduced glutathione (GSH), superoxide dismutase (SOD), and catalase (CAT). A developed self-nanoemulsyfying drug delivery system might be beneficially utilized to improve the oral bioavailability of fisetin and, thus, its neuroprotective activity in vivo [48].

Other researchers prepared traditional nanoemulsions loaded with fisetin consisting of Miglyol 812N, Labrasol, Tween 80, Lipoid E80, and water. The oil droplet size was 153 ± 2 nm. The developed nanoemulsion carrier was subjected to pharmacokinetic studies in mice. After intraperitoneal administration, a 24-fold increase in fisetin bioavailability occurred compared to treatment with fisetin alone. However, there was no such effect after i.v. injection where pharmacokinetic parameters were similar for free and encapsulated fisetin. This can be explained by the faster absorption of fisetin from nanoemulsion formulation and lymphatic distribution. The intravenously administered nanoemulsion had an almost eight-fold greater AUC value than fisetin-loaded SNEDDS administered orally [48]. The antitumor activity of the fisetin nanoemulsion was evaluated on Lewis lung carcinoma-bearing mice. A relatively low dose of fisetin formulation (36.6 mg/kg) was needed to reduce the tumor size by 53% compared to free fisetin (223 mg/kg). As shown in the study, fisetin’s bioavailability and antitumor efficacy can be increased via the formulation of nanoemulsion [49].

### 4.5. Lipid Structures

#### 4.5.1. Solid Lipid Nanoparticles

**Figure 4:**
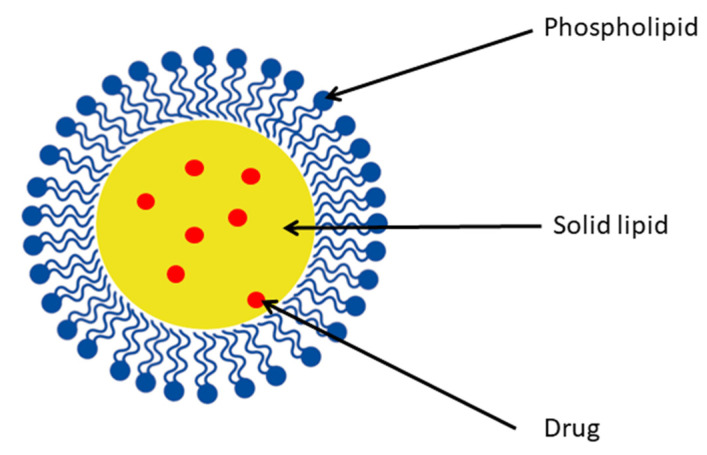
Structure of solid lipid nanoparticle.

The history of solid lipid nanoparticles (SLNs) dates back to the 90s when three independent research groups performed the first experiments to produce solid lipid microspheres [50]. To overcome their drawbacks, they were created as alternatives to liposomes and polymeric nanoparticles. SLNs are colloidal carrier systems often prepared via high-pressure homogenization of lipids, emulsifiers, and water. Their particle size is between <100 and 1000 nm. Their structure is depicted in Figure 4. The lipophilic matrix remains solid at room and body temperature. This provides controlled release and protection for labile active molecules. They are synthesized using physiological lipids with the avoidance of organic solvents in order to reduce toxicity. As a delivery system, SLN offers many potential advantages, such as better bioavailability, especially for lipophilic drugs [51,52]. Considering this benefit, the researchers chose SLN as a carrier for fisetin to improve its biological effect.

Kulbacka et al. experimented with a multifunctional delivery system combining therapeutic and diagnostic functions. They designed solid lipid nanoparticles loaded with fisetin and photosensitizer IR-780 (in a 5:1 ratio) using the solvent diffusion method. Phospholipon 90 G, cetyl palmitate, and Tween 80 were used to fabricate SLNs, exhibiting a hydrodynamic diameter of 133.8 nm and fisetin encapsulation efficiency of 41.19%. Biological studies were performed on hamster ovarian fibroblastoids (CHO-K1) and human colon adenocarcinoma (LoVo) cell lines pretreated with electropermabilization. After electroporation, the prepared LoVo cells were incubated with fisetin-IR-780 co-loaded SLN for photodynamic reaction. Irradiation was carried out for 10 min or 4 h using light with a wavelength in the 750–780 nm range. After 24 hours, cell viability showed a significant decrease (ca. 50%) and was maintained even after 72 hours. Treatment with fisetin-IR-780 SLN formulation caused cell cytoskeletal reorganization and increased p53 and manganese superoxide dismutase expression. These results show that combined therapy, consisting of photodynamic treatment, electroporation, and SLNs, effectively delivers photosensitizer and anticancer drugs. Such multifunctional nanocarriers are promising for detecting and treating colon cancer [53].

#### 4.5.2. Liposomes

Liposomes are self-assembled, spherical vesicles. They are composed of natural or synthetic lipids and are used to form one or more lipid bilayers. The polar head groups are oriented to inner and outer space, creating a hydrophilic core [54]. Their amphiphilic nature enables the incorporation of both hydrophobic and hydrophilic compounds in different parts of the vesicle. The liposomal formulation can be prepared to have various functionalities depending on factors such as selection of phospholipids, number of lipid bilayers, size, charge, and attached modifying groups on the surface. They can be designed in a way that is appropriate for many administration routes, such as oral, parenteral, and topical [55,56,57,58]. Based on the number of bilayers, liposomes can be divided into multivesicular, multilamellar, and unilamellar vesicles. Additionally, the latter can be classified as small (20–100 nm), large (>100 nm), and giant (>1000 nm) [59,60]. They can be classified into different types such as ethosomes [61], binary ethosomes [62], transethosomes [63], niosomes [64], glycosomes [65], and others [66,67] based on the modifications in their lipid membrane composition. Changes in the structure of lipid bilayer lead to improved stability or skin permeability compared to traditional liposomes [68]. They enhance solubility, protect encapsulated molecules, and provide the controlled release of drugs [69]. For this reason, liposomes have become extremely popular as drug delivery systems for bioactive compounds. In terms of toxicity, they are relatively safe. However, there is evidence of DNA damage in the spleen and lungs after the i.v. injection of cationic liposomes [70,71]. Nevertheless, many liposomal drug products have been approved by the U.S. Food and Drug Administration (FDA) and European Medicines Agency (EMA) for use in cancer therapy [72,73], infections [74], vaccines [75], anesthesia [76], and photodynamic therapy [77]. According to the literature reports on the subject, polyphenols such as fisetin were also encapsulated [78]. Table 2 summarizes the liposome types, preparation methods, characterization, size, entrapment efficiency, and in vitro and in vivo studies of fisetin complexes. There is a particular focus on improving fisetin bioavailability for topical application.

Moolakkadath et al. prepared fisetin-loaded transethosomes with good entrapment efficiency of 68.31 ± 1.48%. The developed formulation was subjected to Franz cell in vitro skin permeation study using rat skin. Thermoanalytical analysis showed that the prepared formulation made the rat’s skin more fluidized, which enhances the smoothness of fisetin transethosome penetration. Transethosomes composed of phospholipids, ethanol, and surfactant are highly flexible and can squeeze through the pores and penetrate the skin [79,80]. To avoid the removal of the formulation from the skin due to insufficient viscosity, a gel was prepared using Carbopol 934 as a gelling agent. The deramtokinetic study showed that fisetin-loaded transethosome gel achieved better penetration than conventional fisetin gel [81].

Moolakkadath et al. also prepared a gel with fisetin-loaded glycerosomes with an entrapment efficiency of 86.41 ± 2.95% [82]. The skin penetration studies were conducted on rat skin using Franz diffusion cell. The DSC and FTIR analysis revealed the increased water content in the connective dermis layer. This could have been due to the presence of glycerol in the glycerosome vesicles, which induces the hydration and lipid fluidization of the skin layers. Consequently, it facilitates the diffusion of the formulation. Other studies also confirm that the greater fluidity of glycerosomes makes them capable of penetrating deeper skin layers or brain tissue [83,84].

Knowing that liposomal formulations containing fisetin have good skin penetration, it became obvious of the need to examine their biological action against skin cancer. The same research group loaded fisetin in binary ethosomes, with the subsequent formation of a gel [85]. The encapsulation efficiency (89.23 ± 2.13%) was higher than that observed in the previous experiment. The researchers evaluated the efficacy of the developed formulation in preventing UVB-induced skin cancer in an animal model. The mice’s skin was exposed to UVB radiation in the 290–320 nm range for 10 days at 180 mj/cm^2^. Tumor growth was observed weekly. The concentration of inflammation factors such as TNF-α and IL-1α was then measured in skin homogenate. The obtained result showed a decrease in the level of TNF-α and IL-1α (553 ± 12 pg/mL and 934 ± 15 pg/mL, respectively) for the mice pre-treated with fisetin binary ethosomes gel. The inflammatory response was higher for the mice exposed to UV, with TNF-α and IL-1α concentrations of 768 ± 5 pg/mL and 1325 ± 14 pg/mL. There were fewer incidents of tumors in the group of mice treated with fisetin binary ethosomes gel (49%) compared to mice treated with UV only (96%). The proposed fisetin delivery system can potentially be used against skin cancer.

Other researchers prepared traditional liposomes loaded with fisetin to evaluate its cytotoxicity against endothelial EAhy 926 cells, Lewis lung carcinoma (3LL), and colon tumor CT26 cells. The formulation was prepared using phospholipon^®^ 90 G (P90 G) and 2-dioctadecylcarbamoyl-methoxyacetylamino) acetic acid (ω-methoxy)-polyethylene glycol 2000 ester (DODA-GLY-PEG_2000_). The lipid cake formation technique allowed the creation of vesicles with diameters of 175 nm and an encapsulation efficiency of 73%. After the liposomal encapsulation, fisetin retained the activity of free fisetin. The required concentration values to kill 50% of cells were almost the same for both in 3LL (15.5 µg/mL for free fisetin and 15.0 µg/mL for liposomal form). For liposomal formulation in CT26 and EAhy 926 cell lines, the IC_50_ values were 16.7 µg/mL and 17.9 µg/mL, respectively. The present results demonstrate that similar cytotoxicity levels may be associated with the rapid release of fisetin from the liposomes [86].

The same research group designed the fisetin liposomal formulation using 1,2-dioleoyl-sn-glycero-3-phosphocholine (DOPC) and DODA-PEG_2000_ via a thin-film hydration technique. Liposomes had a 173.5 ± 2.4 nm diameter and slightly lower encapsulation efficacy (58%). The anticancer potential of fisetin loaded in liposomes was evaluated in Lewis lung carcinoma (LLC)-bearing mice. After the i.p. administration of fisetin liposomes (21 mg/kg), tumor growth was delayed (3.3 days) compared to fisetin alone (1.6 days). A 47-fold increase in bioavailability was observed after the i.p. administration of encapsulated fisetin compared to free molecule [87].

This research group also investigated the co-encapsulation of fisetin and the anti-cancer medication cisplatin into liposomes. DOPC, cholesterol, and DODA-GLY-PEG_2000_ were used to make the liposomal formulation, which had a diameter of 173 ± 8 nm. The encapsulation efficiency depended on an initial ratio of fisetin and 100% was encapsulated for 1 and 2 wt% of the initial ratio. It was observed that a higher initial ratio of incorporated fisetin (3.2 wt%) led to a decrease in encapsulation to 90%. The antiangiogenic and cytotoxic activity of the designed liposomal formulation was assayed in vitro using human umbilical vein endothelial cell line EA.hy926 and U-87 MG cells. 

Due to the cytotoxicity of cisplatin for EA.hy926 cells, antiangiogenic activity was tested on fisetin-loaded liposomes. The toxic activity of encapsulated fisetin increased, as can be seen from the lower concentration needed to kill 50% of the cells (IC_50_) after 24 and 48 h (135 ± 10 and 99 ± 8 µM), compared to free fisetin (183 ± 18 and 131 ± 6 µM). The cytotoxic effect on glioblastoma cell U-87 MG was evaluated for co-encapsulated fisetin and cisplatin. The liposomal formulation showed an additive effect with an IC_50_ value of 190 ± 103 µM after 24 h. In comparison, the IC_50_ for cisplatin-loaded liposomes and fisetin-loaded liposomes was 58 ± 23 µM and 366 ± 305 µM, respectively [88]. The obtained benefits in the form of improved solubility of fisetin and the combination of antiangiogenic and cytotoxic activity seem to be promising and worth investigating in further in vivo experiments.

**Table 2:** Summary of fisetin liposome formulations, its characterization, and in vitro and in vivo studies.

Lipid Vesicle	Methods of Preparation	Diameter (nm)	Entrapment Efficiency (%)	In Vitro Study	In Vivo Study	Ref.
Liposome (P90 G, DODA-GLY-PEG_2000_)	Lipid cake formation	175	73	3LL, CT26, EAhy 926 cell line	-	[86]
Liposome (DOPC, DODA-PEG_2000_)	Thin-film hydration	173.5 ± 2.4	58	LLC tumor cells—assessment of apoptosis	Anticancer activity (LLC tumor in mice (i.p.))	[87]
Liposome (DOPC, DODA-GLY-PEG_2000_)	Thin-film hydration (cisplatin co-encapsulation)	173 ± 8	90–100	EA.hy926, U-87 MG cell lines	-	[88]
Transethosome (Lipoid S 100, sodium cholate)	Thin-film hydration	74.21 ± 2.65	68.31 ± 1.48	-	Rat skin permeation	[81]
Ethosome (P90 G, EtOH, GLY)	Thin-film hydration	99.89 ± 3.24	89.23 ± 2.13	DPPH assay, TNF-α, IL-1α, lipid peroxidation assay, glutathione assay, catalase enzyme assay	Rat skin permeation	[85]
Glycerosome (Lipoid S 100, GLY)	Thin-film hydration	138.8 ± 4.09	86.41 ± 2.95	-	Rat skin permeation	[82]

Legend: 2-dioctadecylcarbamoyl-methoxyacetylamino) acetic acid (ω-methoxy)-polyethylene glycol 2000 ester (DODA-GLY-PEG_2000_), 1,2-dioleoyl-sn-glycero-3-phosphocholine (DOPC), ethanol (EtOH), glycol (GLY), interleukin-1α (IL-1α), Lewis lung carcinoma (LLC), phospholipon^®^ 90 G (P90 G), tumor necrosis factor-alpha (TNF-α).

#### 4.5.3. Spherulites

Spherulites represent a different type of multilamellar liposome with an onion-like shape. Their highly ordered lamellar structure is obtained through controlled shearing with mechanical force. Lecithin, co-surfactant, cholesterol, or poly(ethylene)glycol are used to synthesize spherulites. The concentric phospholipid bilayers maintain the advantages of liposomes and, at the same time, provide better stability [89,90]. Crauste-Mancient et al. encapsulated fisetin into spherulites consisting of egg lecithin and polysorbate. The hydrodynamic diameter of the prepared vesicles was 230 ± 20 nm. The observations of cryogenic transmission electron microscopy (cryo-TEM) pictures confirmed many lipidic layers (12–18 layers), representing the potential for higher drug loading. As a result, when comparing the liposomal formulation, spherulitic fisetin had a 5-fold higher entrapment efficiency. The cytotoxicity of the prepared formulation was determined on endothelial (EAhy 926) and tumor cells (3LL). In the cytotoxic study, a 1.4- and 1.7-fold decrease in IC_50_ was observed for endothelial and tumor cells, respectively. Such a result may be due to the delayed release of fisetin from spherulitic lipid bilayers [91].

#### 4.5.4. Nanocochleates

Cochleates were created in response to the disadvantages of other lipid-based nanoparticles. They were first discovered in 1975 and are lipid-based supramolecular assemblies [92]. Their major components are negatively charged phospholipids and divalent or multivalent cations. The presence of divalent cations facilitates the rolling-up of lipid bilayer sheets to form spiral shapes. Although they do not possess an internal aqueous phase, cochleates can incorporate a variety of hydrophilic and hydrophobic molecules. In 1999, nanocochleates were introduced as molecules with sizes between 50 and 100 nm, which makes them desirable for use in applications routes [93,94]. More attention is being given to them due to their advantages such as improved stability, efficient incorporation of guest molecules, slow drug release, protection, and enhanced bioavailability of encapsulated pharmaceuticals [95,96].

Fisetin was incorporated into nanocochleates to improve its therapeutic efficacy. Bothiraja et al. developed a novel fisetin formulation using dimyristoyl phosphatidylcholine (DMPC), cholesterol, and calcium ions. After adding calcium ions, the liposomal vesicles with loaded fisetin formed a nanocochleate structure with a particle size of 275 ± 4 nm and an entrapment efficiency of 84.31 ± 2.52%. Pharmacokinetic research into mice after i.p. administration revealed a 141-fold higher bioavailability than when treated with free fisetin. A higher plasma concentration for fisetin nanocochleate was also observed, which could possibly be associated with fisetin’s fast absorption, slow release, and prolonged circulation time. Additional peaks in the HPLC chromatogram may correspond to free fisetin metabolites. The slower clearance indicates that nanocochleate protects fisetin and, thus, is less prone to undergo metabolization. In an in vitro anticancer test on human breast cancer MCF-7 cell line, fisetin nanocochleates showed 1.3-fold-improved growth inhibition in 50% of cells versus fisetin alone. The results underline the value of nanochochleates as carriers of fisetin, improving its bioavailability and anticancer efficacy [97].

## 5. Nanocrystals

Nanocrystals are aggregates of molecules that form the crystalline structure of an active compound coated with a surfactant. Their size is in the nanometer range below 1 µm. Their characteristic feature is a lack of carrier material, meaning that they have only active-compound nanocrystals. Their dissolution in aqueous or non-aqueous media leads to the formation of nanosuspension, which must be stabilized using a surfactant or stabilizer. There are various methods of creating nanocrystals: milling, precipitation, homogenization, and spray-drying [98]. Nanotechnology will affect our lives tremendously over the next decade in different fields, including medicine and pharmacy. Nanocrystals do not belong to this future; the first products are already on the market. Nanodimensioning alters a material’s physical properties, and this can be applied to materials such as nanocrystals and utilized in pharmaceutics to produce a novel creative formulation strategy for poorly soluble pharmaceuticals. The transfer into the nanodimension is beneficial as it increases poorly soluble compounds’ surface area and dissolution velocity. Thus, absorption from the gastrointestinal tract into the bloodstream can be improved [99].

Dzakwan et al. observed that reducing the size of fisetin improves its solubility. They prepared fisetin crystalline nanosuspensions using a precipitation process. The particle size depended on the type of stabilizer used and ranged from 225.7 to 1019.1 nm. The following pattern was observed: polysorbate 80 < sodium lauryl sulfate (SLS) < hydroxypropyl cellulose (HPC) < hydroxypropylmethylcellulose (HPMC) < Eudragit. Reductions in particle size to the nanometer scale increased the solubility of fisetin nanocrystals (420 µg/mL) compared to free fisetin (60.57 µg/mL). This phenomenon is due to the increase in the surface area of the fisetin particles [100].

Other researchers have shown that the use of fisetin nanocrystals improves anticancer effects. Ma et al. designed fisetin nanocrystals via a solvent/antisolvent precipitation process known as the bottom-up method. The prepared formulation had a diameter between 148.6 ± 1.1 and 134.9 ± 1.4 nm. The DSC, NMR, and FTIR results confirmed the nanocrystalline state of fisetin coated with the poloxamer. In in vitro studies after 24 h and 72 h, the concentration required to kill 50% of the 3LL cells was almost the same for fisetin in nanocrystals and free molecules. However, after 72 h, a slight decrease in IC_50_ was observed. Due to the impossibility of evaluating the IC_50_ of free fisetin for EA.hy926 cells, the IC_85_ was calculated. For both incubation times, 4.8- and 5.6-fold decreases in the IC_85_ were visible in the case of fisetin nanocrystals. The effects indicate an improved anticancer activity when fisetin is formulated as nanocrystals. Morphological studies confirmed an increase in the apoptosis of the endothelial cells as well as an improved anti-angiogenic effect of fisetin nanocrystals. The nanosuspension with fisetin was also stable after adding cryoprotectant for 120 and 30 days at −80 °C and 5 °C, respectively [101].

## 6. Biotechnological Modifications and Combination with Biotechnologically Obtained Structures

### 6.1. Complex with Cyclodextrin

Cyclodextrins (CDs) are cyclic oligosaccharides. The most popular and widely used are α-, β- and γ-cyclodextrins made up of six, seven, or eight α-D-glucopyranose subunits, respectively. They are linked by α-1,4-glycosidic bonds. Their end-to-end linkage of glucose subunits results in a toroid structure resembling a truncated cone. They possess a hydrophobic interior and a hydrophilic exterior, with the primary and secondary OH groups pointing to the narrow and wide sides of the cavity, respectively. Because the OH groups face outwards, they are soluble in water and serve as an ideal carrier for lipophilic molecules, improving their solubility and bioavailability [102].

The so-called natural cyclodextrins have solubility limits (measured at 25 °C) that increase in the following order: β-CD (1.85 g/100 mL), α-CD (14.5 g/100 mL), and γ-CD (23.2 g/100 mL) [103]. It is preferable to use derivatives of β-CD with substituted hydroxyl groups, e.g., the sulfobutylether (SBE-β-CD), the hydroxypropyl derivative (HP-β-CD), and the randomly methylated β-CD (RAMEB) [104]. Not only can we customize CDs with the desired solubility for our application, but we can also customize the core to a desired size. The cavity diameter depends on the number of glucose units and can be adjusted to the shape and size of the guest molecule in order to form inclusion complexes. The inner diameters are as follows: α-CD (4.7–5.3 Å), β-CD (6.0–6.5 Å), and γ-CD (7.5–8.3 Å) [103]. This can be even bigger for large-ring cyclodextrins (>8 glucose units), which are suitable as carriers for large guest compounds [105].

Inclusion methods are relatively easy and cost-effective. The most used techniques include co-dissolution, kneading, spray drying, and the supercritical carbon dioxide method [106]. In the case of fisetin complexes with CDs, co-dissolution is the preferred method of inclusion [107,108,109].

It is worth mentioning that, based on many toxicity studies, CDs are generally recognized as safe (GRAS). In particular, γ-CD is well tolerated and without adverse effects [110,111,112,113]. In Table 3, we summarize the methods of preparation and characterization of the complexes, as well as the means of enhancing their solubility enhancement and stability.

**Figure 5:**
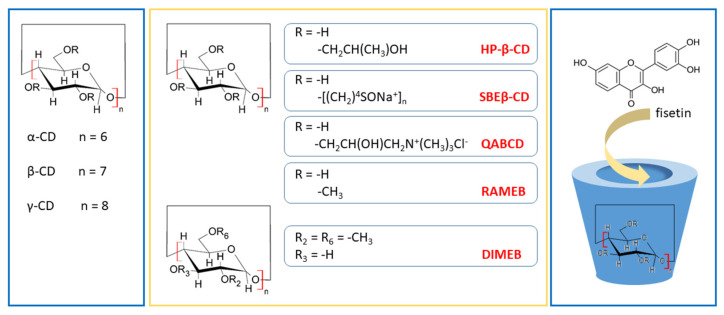
Examples of several types of cyclodextrins used as fisetin carriers. Heptakis-(2,6-di-O-methyl)-β-cyclodextrin (DIMEB), 2-Hydroxypropyl-β-cyclodextrin (HP-β-CD), quaternary ammonium β-cyclodextrin (QABCD), randomly methylated β-cyclodextrin (RAMEB), sulfobutylether β-cyclodextrin (SBE-β-CD).

Fisetin was complexed with various CDs such as natural α-CD, β-CD, and γ-CD, and also their derivatives HP-β-CD, HP-γ-CD, SBEβ-CD, succinyl-2-hydroxypropyl β-cyclodextrin (SHPβ-CD), heptakis-(2,6-di-O-methyl)-β-cyclodextrin (DIMEB), RAMEB, and quaternary ammonium β-cyclodextrin (QABCD) according to the literature (Figure 5). A solubility enhancement from 1.6-fold to 161.9-fold is observed for prepared inclusions. However, not every research group has measured this parameter.

Pahari et al. indicated that flavonols with many hydroxyl groups show enhanced solubility in an aqueous environment in the presence of CDs. This can be explained by the greater interaction of hydroxyl groups with moieties inside the CD core through hydrogen bond formation. The greater the surface of interaction is, the more fisetin molecules will fit inside the lipophilic central cavity. This leads to an increase in solubility [109]. The most soluble fisetin inclusion complex was obtained using the CD derivative—HP-β-CD in PLGA nanoparticles [114]. When comparing β-CD and γ-CD, the solubility of the latter was better. FTIR and NMR results show that γ-CD is compatible with the size of fisetin compound that can improve its solubility [115].

Another important parameter describing the association of CDs with guest molecules is the stability constant. In the case of fisetin with CDs, it was observed in the range of 0.628 to 2309.13 M^−1^. This depends on the temperature and the CD derivative used. Fisetin was the most strongly associated with SHPβ-CD [107]. We can select the most appropriate CD derivative for our application by considering the differences between the binding forces of the examined fisetin CD complexes. 

The prepared complexes with fisetin were used in an in vitro study. Their antioxidant and cytotoxic effects on cancer cells were evaluated. Sali et al. compared the antiproliferative activity of several fisetin CD complexes on HepG2 liver tumor cells. A significant decrease in ATP and total protein levels was visible for the fisetin-DIMEB complex, probably due to the additive effect. DIMEB (30% decrease in ATP and total protein levels) and fisetin (IC_50_: 100 µM) separately displayed cytotoxicity against HepG2 cells, fisetin-β-CD and fisetin-HP-β-CD did not show a significant decrease in ATP and total protein levels [116].

Similarly, fisetin formulations’ antioxidant and cytotoxic (A549 cell line) activity with SBEβ-CD remained unchanged after inclusion [117]. In the test with A2780, MDA-MB-231, and SiHa cell lines, fisetin was very effective and exhibited an inhibitory activity of 79.25%, 40.84%, and 42.63%, respectively. Inclusion in RAMEB decreased biological activity. However, fisetin acted as a strong antiproliferative agent had a worse performance when combined with CDs [118].

The results of the in vitro study by Zhang et al. were more optimistic. The cytotoxic effect of fisetin with β-CD, γ-CD on MCF-7 and Hela cells was greater than that obtained using either CDs or fisetin alone. This shows that inclusion may positively affect fisetin bioactivity [115]. Kadari et al. prepared a fisetin-HP-β-CD complex to improve fisetin’s solubility and enhance its oral bioavailability by incorporating it into PLGA nanoparticles. The prepared formulation was tested for anti-cancer activity against breast cancer cells MCF-7. The cytotoxicity of developed nanoformulations was 3.9 times higher than that of the pure fisetin [114].

As can be seen from the examples, incorporating fisetin into different types of CDs affects their antioxidant and cytotoxic properties. These changes may cause improvement to or the deterioration of bioactivity and can affect the impact the lack of CDs has on fisetin. This can be influenced by factors such as the selected type of CD, the degree of fisetin solubility increase, or the strength of the formed complex.

**Table 3:** Summary of fisetin formulations with cyclodextrins, preparation methods, stability, in vitro study, and influence of cyclodextrins inclusion complexation on solubility.

CD Used with Fisetin	Method(s) of Preparation/Fisetin/CD Ratio	In Vitro Study	Stability Constant (M^−1^)	Solubility Enhancement	Ref.
γ-CD	Co-solvent (water/ethanol)	-	1.46 × 10^3^	-	[108]
HP-γ-CD	Co-solvent (water/ethanol)	-	82.3	No measurable data	[109]
γ-CD	Co-solvent (water/ethanol) then Freeze-drying 1:1	DPPH assay	-	-	[119]
β-CD, HP-β-CD, DIMEB, QABCD	-	HepG2 cell line	3.18 3.63 3.77 4.06 (25 °C)	-	[116]
β-CD, γ-CD	Co-solvent (water/ethanol) then freeze-drying 1:1	Hela and MCF-7 cell line	-	1.6 fold 2.78 fold	[115]
β-CD, SHPβ-CD	Co-solvent (water/methanol)	-	622.56 2309.13 (25 °C)	-	[107]
β-CD, HP-β-CD, SBEβ-CD	Aqueous method; co-solvent (water/ethanol) 1:1	DPPH assay, A549 cell line	0.628 2.291 4.474 (37 °C)	-	[117]
HP-β-CD in PLGA NPs	Coacervation (water/ethanol) 1:1	ROS assay, MCF-7 cell line	1296.03 (37 °C) For FIS-HP-β-CD	161.9 fold A_L_ type	[114]
β-CD, HP-β-CD, RAMEB	Powder mixing; kneading (water/ethanol) 1:2	A2780, MDA-MB-231, SiHa cell line	-	-	[118]
α-CD, β-CD	Co-solvent (water/methanol) 1:1	-	1000 (15 °C) 860 (25 °C) 510 (35 °C) 360 (45 °C) For FIS-β-CD	-	[120]

Legend: α-cyclodextrin (α-CD), β-cyclodextrin (β-CD), γ-cyclodextrin (γ-CD), heptakis-(2,6-di-O-methyl)-β-cyclodextrin (DIMEB), fisetin β-cyclodextrin complex (FIS-β-CD), fisetin 2-Hydroxypropyl-β-cyclodextrin complex (FIS-HP-β-CD), 2-Hydroxypropyl-β-cyclodextrin (HP-β-CD), 2-Hydroxypropyl-γ-cyclodextrin (HP-γ-CD), poly(lactic-co-glycolic acid nanoparticles (PLGA NPs), quaternary ammonium β-cyclodextrin (QABCD), randomly methylated β-cyclodextrin (RAMEB), sulfobutylether β-cyclodextrin (SBEβ-CD), succinyl-2-hydroxypropyl β-cyclodextrin (SHPβ-CD).

### 6.2. Probiotic Bacteria as Vehicle

Osmoporation is a method of obtaining bacterial or yeast-cell-based carriers. This technique takes advantage of the cell’s ability to transfer water through the membrane both ways, moving from intracellular to extracellular media. The subsequent dehydration, hyperosmotic perturbation, and rehydration can lead to the encapsulation of bioactive compounds. This is a new perspective that can protect labile hydrophobic compounds and increase their bioaccessibility [121].

The osmoporation-produced biocapsules using *Lactobacillus acidophilus* were used to encapsulate fisetin. The researchers evaluated the gastrointestinal digestion of the prepared biocapsules. Fisetin-loaded *L. acidophilus* cells were more stable than free fisetin in oral, gastric, and intestinal environments and remained at 69.7%, 29.9%, and 0.7% of the initial fisetin content. During the gastric stage, 99.6% of encapsulated fisetin was retained, while only 45.5% was released during the intestinal stage. The results indicate an increase in the bioavailability and stability of fisetin and create an avenue for further research into the influence of the cell envelope on the mechanism of drug release and digestion [122].

### 6.3. Prebiotic Carbohydrates as Vehicles

Prebiotic carbohydrates such as inulin belong to a class of non-digestible oligosaccharides. They can serve as efficient bioactive delivery systems due to their well-known advantages, i.e., bioavailability, safety, biocompatibility, and low cost of production. As delivery systems, prebiotics possess the beneficial properties of carbohydrates and nanodimensional materials [123,124]. Thus, they have already been applied as material for encapsulating and protecting bioactive compounds and medicinal substances [125,126].

Using prebiotic carbohydrates as vehicles improves the solubility of fisetin, as proven by Charoenwongpaiboon et al. They encapsulated fisetin in inulin nanoparticles by mixing two solutions and performing filtering through a 0.2 µm filter. For this reaction, inulin was synthesized enzymatically from sucrose using inulosucrase from *Lactobacillus reuteri* 121. The size of the inulin molecules depended on the temperature applied during the synthesis and ranged from 95.9 to 238.5 nm at 50 °C and 4 °C, respectively. The encapsulation of fisetin increased its solubility 3.6 times compared with the free compound.

The researchers evaluated the bioefficacy of the fisetin complex with inulin using the DPPH assay. The antioxidant activity was higher compared to the use of fisetin alone. Besides the solubility enhancement, inulin increased the stability of fisetin. During the incubation at 70 °C for 120 min, the complex retained a higher activity of scavenging DPPH radicals than the free fisetin. Thus, combining bioactive compounds with inulin nanoparticles can also lead to better protection from harsh conditions [127].

### 6.4. Glycosylation Method

**Figure 6:**
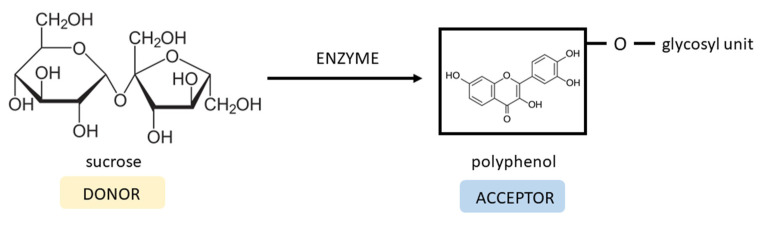
Enzyme-mediated glycosylation of polyphenol with formation of glucoside.

Enzymatic transglycosylation is one of the methods utilized to improve the polarity and solubility of hydrophobic compounds. The glycosyltransferases, e.g., dextransucrase produced by *Lactobacillus*, *Leuconostoc*, and *Streptococcus* species transfer a glycosyl unit from sucrose (donor) to the compound containing a hydroxyl group (acceptor) with the formation of glucosides (Figure 6). The position of transglycosylation on an acceptor molecule depends on the specificity of the enzyme used and can lead to differently functionalized products [128,129]. Scientific studies showed that the attachment of sugar moiety can influence the biological activity of many synthetic and natural compounds [130,131,132].

Moon et al. performed a transglycosylation reaction using dextransucrase from *L. mesenteroides* NRRL B-1299CB4, sucrose, and fisetin. As a result, fisetin-4′-*O*-α-D-glucopyranoside was synthesized. Introducing the glucosyl unit improved the solubility 8.1 times compared to the fisetin compound. Unfortunately, the fisetin derivative reduced antioxidant activity by 25, 28.3, 75, and 215.8% in ORAC and ABTS^•+^ assays at pH 7.4, and in FRAP and ABTS^•+^ assays at pH 3.6, respectively. However, the anti-inflammatory effect of fisetin glucoside was enhanced via the inhibition of NO release by 92.3% compared to fisetin (81.4%). Anti-lipid accumulation activity in mouse 3T3-L1 cells was evaluated, and the results showed similar effects for both compounds [133].

Other researchers obtained even better fisetin solubility by preparing glycoside derivatives of fisetin using cyclodextrin glycosyltransferase (CGTase) from *Paenibacillus* sp. RB01 and β-CD as a glycosyl donor. The HPLC and LC-MS/MS analysis confirmed the synthesis of fisetin mono-, di- and triglucosides and their isomers. Two species of mono glucosides revealed 1800- and 888-times-higher water solubility than fisetin alone. Developed fisetin monoglucosides not only showed enhanced solubility but also maintained antioxidant activity. The concentrations required to scavenge 50% of DPPH radical were comparable for fisetin-7-O-glucoside (2.28 µM), fisetin-4′-O-glucoside (2.52 µM) and fisetin (2.72 µM). Transglycosylation did not affect antioxidant activity in this case, unlike in the previous example [134].

These two studies show that incorporating glucosyl moiety into the fisetin compound significantly improves its solubility, but further evaluation of fisetin derivative properties is needed.

## 7. Comparison of the Effects of Fisetin Pre-Formulation Modifications on the Bioavailability and Bioefficacy Based on In Vivo and In Vitro Models

Although several combinations with carriers have demonstrated encouraging improvements in bioavailability and pharmacokinetic characteristics, it might be challenging to compare them due to variations in delivery methods, starting doses, or experimental design. Table 4 provides an overview of the AUC improvement by fisetin formulations compared to free fisetin. From this data, it can be summarized that nanodelivery formulations have great potential for improving the bioavailability of fisetin, especially through i.p. administration. In many circumstances, it is still important to evaluate therapeutic effectiveness. There has not been much research into enhancing the bioavailability of oral and topical formulations. However, when given intraperitonealy, nanocochleate demonstrated the greatest increase in AUC when compared to free fisetin.

Better bioavailability should result in measurable biological effects. The effects of fisetin and drug delivery systems on cell lines are shown in Table 5. Based on the half-maximal inhibitory concentration (IC_50_) values, it can be concluded that fisetin with polymeric micelles conjugated with folic acid is the most cytotoxic against breast cancer cell line MCF-7 [40]. Also, satisfactory results were obtained using fisetin with polymeric micelles PLA-D-α-tocopheryl-PEG_1000_, where the IC_50_ was 9.68 µg/mL. Additionally, it was demonstrated using in vivo tests that this formulation reduced tumor growth [39]. This suggests that using polymeric micelles could be a successful option for fisetin’s carrier in the battle against breast cancer. On the other hand, fisetin nanocrystals demonstrated maximum cytotoxicity when tested against Lewis lung cancer cells in vitro [101]. In vivo studies showed that fisetin nanoemulsion reduced tumor size and, when incorporated into liposomes (DOPC, DODA-PEG_2000_), delayed tumor growth [49,87]. Again, fisetin nanocrystals were found to be the most effective, this time against the EA.hy926 cell line [101]. This suggests that it may also be useful in treating cardiovascular disorders. Fisetin incorporated into cyclodextrins demonstrated the poorest effect or most serious lack of improvement in cytotoxic activity against cancer cells, indicating that this is not the optimum carrier for enabling fisetin to develop anti-cancer formulations [116,117,118].

## 8. Conclusions

In recent years, researchers have shown the many beneficial properties of fisetin, along with its potential use in the treatment of many diseases. However, despite fisetin’s pharmacological activity, it is still not approved as a drug, mainly because of its poor water solubility and low bioavailability. Many preparations are available on the market as dietary supplements with unknown safety profiles. Although clinical trials with fisetin are underway for such conditions as osteoarthritis or frailty, according to the US FDA (https://clinicaltrials.gov; accessed on 20 June 2023), there is still a need to perform more tests for a wide range of diseases in order to establish their safety in human beings.

Many attempts have been made to improve fisetin bioavailability and bioefficacy by designing novel delivery systems, as evidenced by the numerous results presented in this review. Nanotechnology could deliver fisetin more efficiently for use in various applications. This work reviews the possible influence of nanocarriers and their structural modification on fisetin’s solubility and activity. Despite the large number of studies that have been carried out, there are still several challenges. However, benefits and conclusions can be deduced:

1. Based on in vitro and in vivo antitumor activity studies, it can be concluded that the prepared formulations showed improved anticancer activity after encapsulation.
2. The fact that the encapsulation caused a slower/controlled release and that the fisetin was released gradually was unquestionably in favor of biological action and may have contributed to a greater outcome.
3. Taking into account the potential beneficial impact of fisetin on keratinocyte damage, there is presently limited study on the topical application of fisetin, with most studies focusing on intravenously or intraperitoneally administered doses. However, the results that have been provided open the possibility of carrying out such experiments.
4. More research might focus on the bioefficacy of fisetin in liposomes on skin cancer cells based on the notion that liposomal formulations have good skin penetration.

In summary, studies have shown that fisetin provides various health benefits. Novel formulations should help to increase its bioavailability, which can then improve its therapeutic efficacy in particular diseases. However, further studies are required to fully understand the potential, stability, and safety of novel fisetin nanodelivery systems.

## Figures and Tables

**Table 1:** Summary of possible mechanisms of fisetin action in various diseases.

Disease	Beneficial Effects	Ref.
Periodontitis	Attenuation of periodontitis in vitro and in vivo due to inhibition of inflammatory reaction via FGFR1/TLR4/NLRP3 inflammasome pathway.	[16]
Hypertrophic scars	In vivo inhibition of stretch-induced profibrotic effect by suppressing the proliferation, activation, and collagen production of fibroblasts. Downregulation of FAK and ERK phosphorylation.	[17]
Oxidative skin damage	Increased viability of human keratinocyte HaCaT cells; reduced production of ROS, NO, PGE_2_, IL-1β, and IL-6; inhibited expression of iNOS and COX-2; and activation of NF-κB.	[18]
Polycystic ovary syndrome (PCOS)	In vivo attenuation of clinical features of PCOS (anti-hyperglycemic, anti-hyperlipidemic, and anti-hyperandrogenic activities) as well as increased activity of antioxidant enzymes, i.e., catalase (CAT), superoxide dismutase (SOD), and glutathione peroxidase (GPx).	[19]
Osteoporosis	In vitro inhibition of osteoclastogenesis and promotion of osteoblastogenesis. Osteogenic activity is stimulated through the GSK-3β/β-catenin signaling pathway.	[20]
Heart disease	In vivo reduced ischemia; upregulated expression of PPAR-γ in heart tissue leading to cardioprotection.	[21]
Diabetic cardiomyopathy	In vivo attenuation of oxidative stress, inflammation, and apoptotic cell death.	[22]
	In vivo hypoglycemic and insulin-sensitizing effect with improved cardiac glucose oxidation, and anti-inflammatory, anti-fibrotic and anti-apoptotic effect.	[23]
Alzheimer’s disease	Decreased accumulation of amyloid beta, BACE-1 expression, and hyperphosphorylation of tau protein at serine 413, activation of p-PI3K, p-Akt (Ser 473), and p-GSK3β (Ser 9) expression, and protection from neuroinflammation.	[24]
High-fat diet-induced nephropathy	Reduction in lipid accumulation in the kidney, suppression of oxidative stress and anti-inflammatory response, and iRhom2/NF-κB signaling pathway inhibition.	[25]
Hypercholesterolemia	The modulation of gene expression involved in cholesterol and bile acid metabolism leads to the reduction in total cholesterol, LDL-cholesterol, and hepatic cholesterol content	[26]

Legend: beta-site amyloid precursor protein cleaving enzyme (BACE-1), catalase (CAT), cyclooxygenase 2 (COX-2), extracellular signal-regulated kinases (ERK), focal adhesion kinase (FAK), fibroblast growth factor receptor (FGFR1), glutathione peroxidase (GPx), glycogen synthase kinase *3* beta (GSK-3β), cultured human keratinocyte (HaCaT), inactive rhomboid protein 2 (iRhom2), inducible nitric oxide synthase (iNOS), interleukin-1β (IL-1β), interleukin 6 (IL-6), low-density lipoprotein (LDL), nuclear factor kappa-light-chain-enhancer of activated B cells (NF-κB), the nucleotide-binding oligomerization domain (NOD)-like receptor pyrin domain containing 3 (NLRP3), nitric oxide (NO), polycystic ovary syndrome (PCOS), prostaglandin E2 (PGE_2_), the peroxisome proliferators-activated receptors gamma (PPAR-γ), reactive oxygen species (ROS), superoxide dismutase (SOD), toll-like receptor 4 (TLR4).

**Table 4:** A summary of the novel formulations for improving fisetin’s bioavailability after different routes of administration.

Fisetin Formulation	Route of Administration	Dose	Subject	Fold of AUC Improvement	Ref.
Fisetin-PLA Nanoparticles	i.v.	40 mg/kg	rats	2.32	[32]
Pluronic F127 copolymer conjugated with folic acid (polymeric micelles)	i.p.	15 mg/kg	rats	6.3	[40]
Self-nanoemulsifying drug delivery system (SNEDDS)	oral	20 mg/kg	rats	1.52	[48]
Nanoemulsion (Miglyol 812N/Labrasol/Tween 80/Lipoid E80/water)	i.p.	13 mg/kg	mice	11.92	[49]
Binary ethosomes gel	topical	-	rats	Dermis 4.14 Epidermis 3.11	[85]
Liposomes (DOPC/DODA-PEG_2000_)	i.v. i.p.	13 mg/kg 21 mg/kg	mice	1.64 4.43	[87]
Nanocochleates (DMPC/cholesterol/calcium ions)	i.p.	21 mg/kg	mice	13	[97]

Legend: dimyristoyl phosphatidylcholine (DMPC), dioctadecylamine polyethylene glycol 2000 ester (DODA-PEG_2000_), 1,2-dioleoyl-sn-glycero-3-phosphocholine (DOPC), poly(lactic acid) (PLA), self-nanoemulsifying drug delivery system (SNEDDS).

**Table 5:** A summary of the novel formulations for improving fisetin’s bioefficacy in vitro and in vivo.

Fisetin Formulation	Type of Study	IC_50_ [µg/mL]	Dose [mg/kg]	Cell Culture	Biological Effect	Ref.
Fisetin-PLA nanoparticles	in vivo		40	4T1	Tumor volume reduced to 67%	[32]
	in vitro	29.3		HCT116	Cell viability < 40% (200 µg/mL)	
Fisetin-PVP nanoparticles	in vitro	80		MDA-MB-231	Cell viability reduced to 35%	[34]
Fisetin polymeric micelles conjugated with folic acid	in vitro	4.9		MCF-7	GI_50_ decreased by 65.737%	[40]
Fisetin HSA NPs	in vitro			MCF-7	Stronger cytotoxic effect than free fisetin	[42]
Fisetin polymeric micelles PLA-D-α-tocopheryl-PEG_1000_	in vivo			MCF-7	Tumor growth ↓ Induction of apoptosis after 24 h (20%), 48 h (42%)	[39]
	in vitro	9.68			Increased cytotoxicity	
Fisetin nanocochleates (DMPC/cholesterol/calcium ions)	in vitro	11.2		MCF-7	Increased cytotoxicity	[97]
Fisetin-HP-β-CD incorporated into PLGA	in vitro	22.09		MCF-7	Increased cytotoxicity	[114]
Fisetin-MPEG-PCL polymeric micelles	in vivo (i.v.)		50	SKOV3	Tumor growth inhibition of 70.7%	[38]
	in vitro	13.79			Increased cytotoxicity	
Fisetin complexed with β-CD, HPβ-CD, RAMEB	in vitro			A2780 MDA-MB-231 SiHa	Free fisetin was more cytotoxic than cyclodextrin inclusions	[118]
Fisetin-IR-780 co-loaded SLN	in vitro			LoVo	After 24 h cell viability decreased by ca. 50% Increase in p53 and manganese superoxide dismutase expression	[53]
Fisetin-DIMEB	in vitro			Hep62	ATP ↓, total protein levels ↓ The presence of BCD, HPBCD, or DIMEB did not modify considerably the effects of fisetin	[116]
Fisetin-SBEβ-CD	in vitro	67.97		A549	Antioxidant and cytotoxic activity unchanged after encapsulation	[117]
Fisetin-MPEG-PCL polymeric micelles	in vivo (i.v)		10	LL/2	Volume and weight of the tumor ↓	[33]
	in vitro	16.63			Increased cytotoxicity	
Fisetin nanoemulsion (Miglyol 812N/Labrasol/Tween 80/Lipoid E80/water)	in vivo (i.p.)		36.6	3LL	Reduced tumor size by 53%	[49]
Fisetin in liposome (P90 G, DODA-GLY-PEG_2000_)	in vitro	17.9		EA.hy926	Similar cytotoxicity values as free fisetin	[86]
		15.5		3LL		
		16.7		CT26		
Fisetin in liposome (DOPC, DODA-PEG_2000_)	in vivo (i.p.)		21.0	3LL	Delayed tumor growth of 3.3 days	[87]
Fisetin nanocrystals	in vitro	15.8		EA.hy926	Increased apoptosis	[101]
		13.5		3LL	The same effect as free fisetin on 3LL	
Fisetin spherulites	in vitro		25.1	EA.hy926	Increased cytotoxicity	[91]
			26.8	3LL		
Fisetin liposome (DOPC, DODA-GLY-PEG_2000_)	in vitro	38.64		EA.hy926	Increased cytotoxicity	[88]
		12.59		U-87 MG		
Fisetin-MPEG-PCL polymeric micelles	in vivo (s.c.)		50	CT26	Extended life of mice by 17 days Suppression of the tumor growth	[37]
	In vitro	7.97			Increased cytotoxicity	

Legend: ↓ refers to the decrease, β-cyclodextrin (β-CD), heptakis-(2,6-di-O-methyl)-β-cyclodextrin (DIMEB), dimyristoylphosphatidylcholine (DMPC), dioctadecylamine polyethylene glycol 2000 ester (DODA-PEG_2000_), 1,2-dioleoyl-sn-glycero-3-phosphocholine (DOPC), human serum albumin nanoparticles (HAS NPs) concentration of the agent that inhibits growth by 50% (GI_50_), 2-Hydroxypropyl-β-cyclodextrin (HP-β-CD), half maximal inhibitory concentration (IC_50_), monomethyl poly(ethylene glycol)-poly(ε-caprolactone) (MPEG-PCL), poly(lactic acid) (PLA), poly(lactic-co-glycolic acid (PLGA), polyvinylpyrrolidone (PVP), randomly methylated β-cyclodextrin (RAMEB), sulfobutylether β-cyclodextrin (SBEβ-CD), solid lipid nanoparticles (SLN).

## Data Availability

Not applicable.

## Abbreviations

ABTS	2,2′-azino-bis(3-ethylbenzothiazoline-6-sulfonic acid)
AKT	protein kinase B
AUC	area under the curve
BACE1	beta-site amyloid precursor protein cleaving enzyme
CAT	catalase
α-CD	α-cyclodextrin
β-CD	β-cyclodextrin
γ-CD	γ-cyclodextrin
CDs	cyclodextrins
COX-2	cyclooxygenase 2
CryoTEM	cryogenic transmission electron microscopy
DIMEB	heptakis-(2,6-di-O-methyl)-β-cyclodextrin
DMPC	dimyristoyl phosphatidylcholine
DODA-GLY-PEG_2000_	2-dioctadecylcarbamoyl-methoxyacetylamino) acetic acid (ω-methoxy)-polyethylene glycol 2000 ester
DOPC	1,2-dioleoyl-sn-glycero-3-phosphocholine
DSC	Differential Scanning Calorimetry
EMA	European Medicines Agency
ERK	extracellular signal-regulated kinases
FAK	focal adhesion kinase
FDA	Food and Drug Administration
FGFR1	fibroblast growth factor receptor
FIS-β-CD	fisetin β-cyclodextrin complex
FIS-HP-β-CD	fisetin 2-Hydroxypropyl-β-cyclodextrin complex
FRAP	ferric reducing antioxidant power assay
GSH	reduced glutathione
GSK-3 β	glycogen synthase kinase *3* beta
GPx	glutathione peroxidase
GRAS	generally recognized as safe
HaCaT	Cultured Human Keratinocyte
HP-β-CD	2-Hydroxypropyl-β-cyclodextrin
HPC	hydroxypropyl cellulose
HPMC	hydroxypropyl methylcellulose
HSA-NPs	human serum albumin nanoparticles
IL-1α	interleukin-1α
IL-1β	interleukin-1β
IL-6	interleukin 6
IL-8	interleukin 8
iNOS	inducible nitric oxide synthase
iRhom2	the inactive rhomboid protein 2
LDL	low-density lipoprotein
LL/2	Lewis lung carcinoma cell line
LLC	Lewis lung carcinoma
MAPK	mitogen-activated protein kinases
MPEG-PCL	methoxy poly(ethylene glycol)-poly(ε-caprolactone)
mTOR	serine-threonine protein kinase
mTORC_2_	mammalian target of rapamycin complex 2
NLRP3	the nucleotide-binding oligomerization domain (NOD)-like receptor pyrin domain containing 3
NF-κB	nuclear factor kappa-light-chain-enhancer of activated B cells
NO	nitric oxide
NPs	nanoparticles
Nrf2	nuclear factor erythroid 2-related factor 2
ORAC	oxygen radical absorbance capacity
PCL	poly-(ε-caprolactone)
PCOS	polycystic ovary syndrome
PGE_2_	prostaglandin E2
PI3K	phosphoinositide-3-kinase
PLA	poly(lactic acid)
PLGA	poly(lactic-co-glycolic acid
PLGA-PEG-COOH	poly(D,L-lactic-*co*-glycolic acid)-*block*-poly(ethylene glycol) carboxylic acid
PPAR-γ	the peroxisome proliferators-activated receptors gamma
PTEN	phosphatase and tensin homolog deleted on chromosome 10
PVA	polyvinyl alcohol
PVP	poly(vinylpyrrolidone)
QABCD	quaternary ammonium β-cyclodextrin
RAMEB	randomly methylated β-cyclodextrin
ROS	reactive oxygen species
SBE-β-CD	sulfobutylether β-cyclodextrin
SHPβ-CD	succinyl-2-hydroxypropyl β-cyclodextrin
SLN	solid lipid nanoparticles
SLS	sodium lauryl sulfate
SNEDDS	self-nonoemulsifying drug delivery system
SOD	superoxide dismutase
TLR4	toll-like receptor 4
TNF-α	tumor necrosis factor-alpha
TRAIL	tumor necrosis factor-related apoptosis-inducing ligand
VEGF	vascular endothelial growth factor
